# Successful recovery after major surgery: moving beyond length of stay

**DOI:** 10.1186/2047-0525-3-4

**Published:** 2014-07-08

**Authors:** Timothy E Miller, Monty Mythen

**Affiliations:** 1 Department of Anesthesiology, Duke University Medical Center, Durham, NC 27710, USA; 2 Institute of Sport Exercise and Health, UCLH National Institute of Health Research, Biomedical Research Centre, London, UK

**Keywords:** Enhanced recovery after surgery, Length of stay, Complications, Quality of recovery, Patient experience

## Abstract

There is strong evidence that Enhanced Recovery Pathways improve length of hospital stay, readmission rates, and complications after major surgery. However, recovery is a complex process that only finishes when the patient returns to normal function. Future studies should also address the patient experience, as well as functional recovery and quality of life after major surgery.

## Background

Enhanced Recovery After Surgery (ERAS) or ‘fast-track’ surgery pathways are evidence-based care pathways that have been developed to accelerate recovery after major surgery. A number of randomized controlled trials (RCTs) have demonstrated that Enhanced Recovery Programs (ERPs) can benefit patients undergoing major surgery [1]. There are also a number of Quality Improvement (QI) projects showing similar benefits [2-4], the largest being the implementation of the NHS Enhanced Recovery Pathway in the UK.

Implementation of ERPs is a complex process that affects multiple departments within a hospital, and requires cooperation from surgical teams, management, and procurement. Most implementation cycles take 3 to 6 months, and are therefore not ideally suited to an RCT. As the benefits of ERPs seem consistent and robust, most of the new studies reporting benefits are likely to be QI projects rather than RCTs.

Published studies to date evaluating the outcome benefit of ERPs have focused on short-term benefits. There is strong evidence that ERPs improve short-term factors such as length of hospital stay, readmission rates, and complications after major surgery [5]. These outcomes are important and need to be studied, but equally important is patient experience and the quality of recovery (QoR) from the patient’s perspective [6]. Patients do not define recovery as being healed physically; instead they define recovery as “the absence of symptoms and return of their ability to perform activities as they could prior to surgery” [7].

Recovery after anesthesia and surgery is a complex process dependent on patient, surgical, and anesthetic characteristics, as well as presence of any of numerous adverse sequelae [8]. It involves multiple domains, including physical, psychological, and social aspects [9]. There is no standard definition or measure. The natural trajectory of recovery after major surgery is characterized by a period of immediate deterioration post-surgery, followed by a gradual rehabilitation to baseline level [9]. This rehabilitation period can last much longer than healthcare providers expect. In a study of patients aged over 60 years undergoing elective abdominal surgery, less than 50% of patients had recovered to baseline levels of physical performance at 6 months after surgery, and 20% of patients were still unable to perform basic daily activities of living [10]. More recently, fewer than 60% of patients within an ERP had returned to baseline walking capacity at 3 months after elective colorectal surgery [11].

Archer *et al*. recently published an in depth analysis in *Perioperative Medicine*, exploring the experience of a small number of patients undergoing gynaecological surgery within an ERP [12]. The patients undertook a semi-structured interview after they were discharged from hospital, in order to explore the overall experience during their hospital stay. This approach is unique in that it gives an in-depth view of participation in an ERP from a patient perspective.

The results are fascinating, and reinforce the fact that the traditional approach to major surgery is as ingrained in patients as it was in many medical staff before the advent of ERPs. George Bernard Shaw once quoted: “I enjoy convalescence: it’s the part that makes the illness worthwhile”. In many ways, this is true; patients expect to be resting and recuperating after major surgery.

The patients studied were particularly concerned about early mobilization for a number of reasons: pain, the presence of catheter and drips, and fear of damaging the surgical wound. The presence of a physiotherapist was integral in alleviating these fears, and importantly, once they were out of bed, patients found that being mobile was not as difficult as they expected.

There is no doubt that bed rest is detrimental to patient recovery. This is not just true for surgical patients but for all patients in hospital. At least 30% of patients aged over 70 years and hospitalized for a medical illness are discharged with a new hospitalization-associated disability; that is, the loss of ability, by discharge, to perform one of the basic activities of daily living, such as showering, dressing, eating, walking without assistance and transferring out of a chair [13]. This is an astounding figure, with profound implications for these patients and society, and iatrogenic factors such as prolonged bed rest are thought to contribute significantly [14]. The onus is on hospitals and healthcare systems to invest in physiotherapy and occupational therapy so that all patients can be intensively mobilized as needed during their hospital stay. This investment will almost certainly be rewarded with improvements in recovery and cost savings in the long term.

The other major finding by Archer *et al*. was that patients wanted to go home, even if they needed extra support, as they related home to normality. However, the home environment has additional challenges, and communication with the hospital is important. Again, this is not surprising, and reinforces the concept that in the future, the model of healthcare may be very different, with more emphasis on patient support, both in and out of hospital, to enable earlier discharge and aid recovery.

This type of research is very informative, and can help assess the impact of changes in healthcare delivery. To aid further research, a postoperative quality of recovery score, the QoR-15, has recently been developed, which can provide an extensive yet efficient evaluation of postoperative recovery from the patient’s perspective [8]. The QoR-15 is an abbreviation of the longer and more comprehensive QoR-40 [15], and includes questions on pain, physical comfort, physical independence, psychological support and emotional state, and can be completed in approximately 2 minutes. Future research should focus on developing and validating instruments such as the QoR-15 for the context of recovery within specific populations of patients. Furthermore, clinically relevant changes in any quality of recovery score need to be determined, so that studies aiming to improve the quality of recovery can be adequately powered to detect a meaningful and clinically relevant change [9].

## Conclusion

As ERPs become the new standard of care and best practice, future studies are likely to concentrate more on the patient perspective, with return to normal function considered the benchmark for recovery after major surgery. Further research into the experiences of all patients within the unfamiliar hospital environment will help hospitals and healthcare services to deliver the care that is needed to improve outcomes.

## Abbreviations

ERAS: Enhanced Recovery After Surgery; ERP: Enhanced Recovery Project; NHS: National Health Services; QI: Quality Improvement; QoR: Quality of Recovery; RCT: Randomized controlled trial.

## Competing interests

MM was National Clinical Lead, UK Department of Health Enhanced Recovery Partnership until March 2013, and is now a National Clinical Advisor and the Editor-in-chief, of *Perioperative Medicine*. TEM has no relevant competing interests.

## Authors’ contributions

TEM and MM were involved in the preparation of the manuscript. Both authors read and approved the final manuscript.
